# Modulation of γ-Secretase Activity by a Carborane-Based Flurbiprofen Analogue

**DOI:** 10.3390/molecules26102843

**Published:** 2021-05-11

**Authors:** Stefan Saretz, Gabriele Basset, Liridona Useini, Markus Laube, Jens Pietzsch, Dijana Drača, Danijela Maksimović-Ivanić, Johannes Trambauer, Harald Steiner, Evamarie Hey-Hawkins

**Affiliations:** 1 Institut für Anorganische Chemie, Universität Leipzig, Johannisallee 29, D-04103 Leipzig, Germany; ssaretz@mannin.de (S.S.); dona_u001@hotmail.com (L.U.); 2 Chemische Biologie, Helmholtz-Zentrum für Infektionsforschung, Inhoffenstraße 7, D-38124 Braunschweig, Germany; 3 Biomedical Center Munich (BMC), Metabolic Biochemistry, Ludwig-Maximilians-University, Feodor-Lynen-Straße 17, D-81377 München, Germany; gabriele.basset@mail03.med.uni-muenchen.de (G.B.); johannes.trambauer@mail03.med.uni-muenchen.de (J.T.); harald.steiner@mail03.med.uni-muenchen.de (H.S.); 4 Department of Radiopharmaceutical and Chemical Biology, Institute of Radiopharmaceutical Cancer Research, Helmholtz-Zentrum Dresden-Rossendorf (HZDR), Bautzner Landstraße 400, D-01328 Dresden, Germany; m.laube@hzdr.de (M.L.); j.pietzsch@hzdr.de (J.P.); 5 Faculty of Chemistry and Food Chemistry, School of Science, Technische Universität Dresden, 01069 Dresden, Germany; 6 Department of Immunology, Institute for Biological Research “Siniša Stanković”, National Institute of Republic of Serbia, University of Belgrade, Bul. Despota Stefana 142, 11060 Belgrade, Serbia; dracadiana@gmail.com (D.D.); nelamax@ibiss.bg.ac.rs (D.M.-I.); 7 German Center for Neurogenerative Diseases (DZNE) Munich, Feodor-Lynen-Straße 17, D-81377 München, Germany

**Keywords:** Alzheimer, carborane, flurbiprofen, γ-secretase modulator (GSM), small molecule, amyloid-β (Aβ) peptide, phenyl mimetic

## Abstract

All over the world, societies are facing rapidly aging populations combined with a growing number of patients suffering from Alzheimer’s disease (AD). One focus in pharmaceutical research to address this issue is on the reduction of the longer amyloid-β (Aβ) fragments in the brain by modulation of γ-secretase, a membrane-bound protease. *R*-Flurbiprofen (tarenflurbil) was studied in this regard but failed to show significant improvement in AD patients in a phase 3 clinical trial. This was mainly attributed to its low ability to cross the blood–brain barrier (BBB). Here, we present the synthesis and in vitro evaluation of a racemic *meta*-carborane analogue of flurbiprofen. By introducing the carborane moiety, the hydrophobicity could be shifted into a more favourable range for the penetration of the blood–brain barrier, evident by a logD_7.4_ value of 2.0. Furthermore, our analogue retained γ-secretase modulator activity in comparison to racemic flurbiprofen in a cell-based assay. These findings demonstrate the potential of carboranes as phenyl mimetics also in AD research.

## 1. Introduction

Alzheimer’s disease (AD) is a neurodegenerative disease, which is characterised by slow but progressive cognitive decline [1]. To this day it is incurable, and current treatments apply acetyl cholinesterase inhibitors or *N*-methyl-d-aspartate receptor antagonists, thereby offering only short-term symptomatic relief [1,2]. At the cellular level, the pathology of AD is characterised by neuritic extracellular plaques and intracellular neurofibrillary tangles [1]. The plaques consist mainly of amyloid-β (Aβ) peptide fibrils. Aβ peptides are produced in different lengths with Aβ38, Aβ40, and Aβ42 being the most abundant species [3]. The longer and more aggregation-prone Aβ42 (and Aβ43) [3] species facilitates Aβ aggregation and is the main component of parenchymal plaques; therefore, increased Aβ42:Aβ40 ratios are believed to be a major trigger of the disease [4]. The different Aβ species are generated by sequential cleavages of the amyloid precursor protein (APP). APP is first processed by β-secretase resulting in a 99 amino acid long C-terminal fragment, which is then further processed within its transmembrane domain by γ-secretase [3]. In this step, γ-secretase produces the different Aβ species by sequential carboxy-terminal trimming of the Aβ48 and Aβ49 species that are initially generated by the first intramembrane cleavage by γ-secretase [3]. This stepwise cleavage activity, also called γ-secretase processivity, is impaired by most inherited AD-causing mutations, resulting in an increased production of more pathogenic, longer Aβ42 and Aβ43 species [3]. Besides its role in APP cleavage, γ-secretase is also important for many other physiological processes such as the Notch signalling pathway, the most prominent example [5].

In order to reduce Aβ plaques in patients, potent γ-secretase inhibitors (GSIs), such as semagacestat, were developed. Unfortunately, semagacestat caused severe side effects, including weight loss, skin cancer and infections, in a phase 3 clinical trial that led to the discontinuation of most GSI study programs [6,7,8]. The side effects are considered to be the result of a mechanism-based toxicity due to the interference with the cleavage of other crucial γ-secretase substrates such as the transmembrane receptor Notch, which is important for nuclear signalling [6].

An alternative to the inhibition of γ-secretase is to modulate its cleavage activity. This concept is based on the findings of Weggen et al. (2001) that certain non-steroidal anti-inflammatory drugs (NSAIDs) lead to a reduced production of Aβ42, thereby lowering the Aβ42:Aβ40 ratio in mouse brain and cell culture [9]. This change in the production ratios was termed γ-secretase modulation, and substances having this profile were called γ-secretase modulators (GSMs) [10,11]. In a follow-up study, Eriksen et al. could show that flurbiprofen displayed the highest potency to lower the Aβ42:Aβ40 ratio in H4 cells and mice [12]. No difference in GSM potency was observed for *S*-, *R*- or racemic flurbiprofen [12]. In order to avoid side effects due to the fact of COX inhibition, only *R*-flurbiprofen (tarenflurbil) was tested in a clinical phase 2 trial. Patients with mild AD showed a significantly slower cognitive decline in comparison to the placebo group. In patients with moderate AD, however, no significant difference was observed [13]. Unfortunately, the previously observed significant effect could not be reproduced in the follow-up phase 3 clinical trial [14]. The failure of *R*-flurbiprofen in phase 3 was attributed to the drug’s poor penetration of the blood–brain barrier (BBB) [15]. Therefore, future developments tried to improve this particular feature of the drugs, e.g., by increasing the compound’s hydrophobicity (logD of 1 to 3, respectively logD ≥ 1.5) [16,17]. Both logD ranges are considered to be beneficial for BBB crossing.

Although it became clear over the past years that the underlying pathomechanism of AD is not sufficiently understood and other factors besides Aβ are increasingly discussed with respect to the development and treatment of the disease, GSMs are still a valid option as part of a combination therapy [2]. Since the failure of *R*-flurbiprofen, constant effort has been made to develop new and more potent GSMs. Most of these next-generation GSMs share an imidazole-aryl moiety, first introduced by the company Eisai [11]. Unfortunately, most of them do not yet combine good in vitro and in vivo properties, resulting in poor solubility in water and potential hepatotoxicity [11]. Therefore, NSAIDs like flurbiprofen and their central structural motif (2-arylpropionic acid) should not yet be fully abandoned for the development of anti-AD drugs.

Among others, Peretto et al. have tried to improve the drug-like properties of this structure. They addressed the metabolically unstable 3′- and 4′-positions at the terminal ring of flurbiprofen, which normally is hydroxylated or methoxylated [18,19,20]. They could show that bulky moieties at these positions, such as 4′-trifluoromethyl, 3′,4′-dichloro or 4′-(4-trifluoromethylcyclohexyloxy) substituents, are favourable for a reduction of Aβ42 production in H4 cells that express APP harbouring the “Swedish” familial AD mutation [20]. Further substitution of the carboxylic acid with known carboxylic acid bio-isosteres, such as tetrazole, in order to minimise plasma protein binding, however, was not beneficial for the compound’s ability to reduce Aβ42. It also resulted in additional interactions of the compound with cytochrome P450 (CYP) isoforms other than CYP2C9 [20]. Furthermore, results from Abdul-Hay et al. suggest that the carboxylic acid group is crucial for flurbiprofen’s GSM activity [21]. Notably, the 4-butylnitrat ester of flurbiprofen, HCT-1026, showed a complete inverse GSM activity profile marked by increasing the Aβ42 levels due to the inhibition of Aβ42 clearance [21,22].

Carboranes (C_2_B_10_H_12_, dicarba-*closo*-dodecaborane(12)s) are frequently used as phenyl mimetics for biologically active compounds [23]. Carboranes are highly hydrophobic, non-toxic and metabolically stable boron clusters in which two BH^−^ vertices are replaced by neutral CH groups. The carbon atoms can be selectively deprotonated by strong bases and functionalised easily. The three different isomers of carboranes, *ortho*-(1,2-dicarba-*closo*-dodecaborane(12)), *meta*-(1,7-dicarba-*closo*-dodecaborane(12)) and *para*-carborane (1,12-dicarba-*closo*-dodecaborane(12)), show increased chemical and biological stability in this order. Thus, *ortho*-carborane can be thermodynamically converted to *meta*- and *para*-carborane at high temperatures [24,25]. Due to the fact of their remarkable stability and hydrophobicity, a growing interest in carboranes for medicinal chemistry applications is becoming evident [26,27,28,29,30,31,32,33,34]. Although the first use of carborane derivatives in boron neutron capture therapy (BNCT) dates back to the 1950s [35], it was only in 2009 that Hawkins et al. showed conclusively that a carborane derivative, namely, a neutral rhenacarborane, can cross the BBB [36]. In 2011, Crossley et al. then showed that both the neutral *ortho*-carborane and the corresponding anionic *nido*-carborane derivative of pyrazolopyrimindines were taken up effectively into T98G human glioma cells [37]. The first carborane-based compound able to affect the central nervous system (CNS) was published in 2014 by Wilkinson et al. [34]. Here, the antidepressant activity of a highly hydrophobic neutral *closo*-carborane derivative (logD_7.4_ = 4.29) was compared with its more hydrophilic monoanionic *nido*-carborane counterpart (logD_7.4_ = 1.44) [34]. In the forced swim test (FST) performed with mice, only the *nido*-compound showed antidepressant activity, proving its ability to cross the BBB in vivo [34]. It was rationalised by Wilkinson et al. that the more favourable logD_7.4_ value of the *nido*-compound (logD_7.4_ < 3) was responsible for crossing the BBB, since the trend of activity between the *ortho*- and *nido*-compounds was reversed when performing in vitro IC_50_ measurements on the targeted P2X_7_ receptor [34].

In this work, we present our approach to replace the terminal phenyl ring of the flurbiprofen scaffold with a bulkier and biologically stable *meta*-carborane moiety to increase the hydrophobicity into a logD_7.4_ range from 1 to 3 for potentially better BBB penetration capabilities while retaining its ability to reduce Aβ42 [23,26].

## 2. Results

### 2.1. Synthesis

**Scheme 1:**
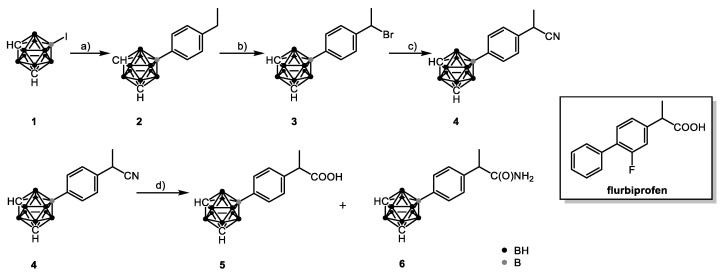
Synthesis of compound **5**. (**a**) 4-Ethylphenylmagnesium bromide, CuI, [PdCl_2_(PPh_3_)_2_], Et_2_O, 85%; (**b**) NBS, hν, CCl_4_; (**c**) SiMe_3_CN, SnCl_4_, CH_2_Cl_2_, 99% (over two steps); (**d**) HCl_conc. aq_, AcOH_conc._, 36% for **5** and 38% of the partially hydrolysed amide **6** (separable by chromatography); all yields were isolated yields.

The *meta*-carborane analogue of flurbiprofen (**5**) was prepared in four steps (Scheme 1). Starting from the 9-iodo-*meta*-carborane (**1**), an ethylphenyl moiety was introduced via Kumada coupling employing freshly prepared 4-ethylphenylmagnesium bromide. After purification, **2** was obtained in good yield. In the next step, an allylic bromination with *N*-bromosuccinimide (NBS) was carried out without any additional radical starter, as such highly reactive compounds lead to complete decomposition of the starting material. The resulting benzylic bromide **3** was used without further purification. The following substitution of the bromide was carried out with excess of tin tetrachloride (SnCl_4_) and not as reported by Reetz et al. in high catalytic amounts [38]. In the final step, the nitrile was hydrolysed in a mixture of glacial acetic acid and concentrated hydrochloric acid leading to the desired product **5** in 36% yield and the partially hydrolysed amide **6** in 38% yield, both as racemic mixtures. Both could be separated by means of normal phase column chromatography. Acidic conditions instead of the more common basic conditions were chosen as a general synthetic strategy suitable not only for *meta*-carborane but also for the more base-labile *ortho*-carborane derivatives, if required.

### 2.2. Determination of COX Inhibition

Since compound **5** is derived from one of the most common unselective cyclooxygenase (COX) inhibitors, its ability to inhibit both isoforms, ovine COX-1 and human COX-2, was determined. *nido*-Indoborin (compound **2** in [39]), a carborane-based derivative of indomethacin, was also tested in the same assay to exclude interference of the carborane moiety (Table 1). No inhibitory activity of **5** was observed in the performed enzyme-based assay (Table 1), indicating that the modification abolished the COX inhibitory activity of the parent compound. Of note was the measured IC_50_ (COX-2) value for *nido*-indoborin that was approximately 18 times higher than the published one determined by an [^14^C]arachidonic acid-based assay using ovine COX-1 and murine COX-2 [39]. This difference might be explained by differences in the assay setup, which notably differs in the applied final arachidonic acid concentration. In both assays, the inhibitors compete with arachidonic acid for binding in the cyclooxygenase active site so that the IC_50_ value is dependent on the affinity of the inhibitor (*K*_i_), the concentration of the substrate ([S]) and the Michaelis constant (K_M_) of the substrate to the enzyme according to the Cheng–Prusoff equation (*K*_i_ = IC_50_/(1 + ([S]/K_M_)) [40]. In the [^14^C]arachidonic acid-based assay [39] that was originally established by Kalgutkar et al., typically a final concentration of 2 µM [^14^C]arachidonic acid is applied [41]. Using a representative K_M_ of 6.5 µM of arachidonic acid to His-tagged COX-2, the calculated affinity of the inhibitor to the enzyme would be *K*_i(COX-2, calculated)_ = 39 nM [42]. In this work, 100 µM arachidonic acid was applied according to the manufacturer’s instructions for the “COX Fluorescent Inhibitor Screening Assay Kit”. Based on the abovementioned discussion, the *K*_i(COX-2, calculated)_ was 56 nM and, hence, comparable to the previously determined binding potency of *nido*-indoborin to COX-2. This highlights the importance to use a well-established standard compound within the assay. In this and previous work, we used celecoxib as the internal standard and experimentally determined an IC_50_(COX-2) value of 38 ± 20 nM (*n* = 6 biological replicates) converting to a *K*_i(COX-2, calculated)_ = 2.3 ± 1.2 nM. This value is in good accordance with the experimentally determined affinity of [^3^H]celecoxib to COX-2 of K_D_ = 2.3 nM determined by Hood et al. [43].

### 2.3. Determination of logD_7.4_

The logD_7.4_ value for compound **5** and *nido*-indoborin was determined by a previously reported HPLC method [44], which was originally described by Donovan and Pescatore [45]. Under standard conditions (method 2, Table 2) the logD_7.4_ of compound **5** was found to be 1.86 and, hence, higher in comparison to flurbiprofen [46]. For *nido*-indoborin, the logD_7.4_ was determined to be 2.05. Of note, applying another column (i.e., Chromolith Speed ROD RP-18E 50 (method 1, Table 2)) instead of the typically used Asahipak ODP-50 4B, in an equal setting resulted in comparable logD_7.4_ values. The measured variances in logD_7.4_ measurements between the two compounds and the two methods lie in the typical range of the method, and both compounds can be seen as equally hydrophobic [45]. This confirms that the replacement of a phenyl ring with a carborane moiety indeed increased the hydrophobicity and could therefore result in an increased BBB penetration of compound **5** compared to flurbiprofen.

### 2.4. Determination of Cytotoxicity

The toxicity of compound **5** up to a concentration of 100 µM for 48 h incubation time was determined in several cell lines by employing the 3-(4,5-dimethylthiazol-2-yl)-2,5-diphenyltetrazolium bromide (MTT) and crystal violet (CV) tests and presented as IC_50_ values (Table 3). The used human tumour (i.e., melanoma A375, colon cancer HCT116 and HT29, and glioblastoma LN229 and U251) and rodent tumour (i.e., mouse melanoma B16 and B16–F10; rat glioma C6) cell lines as well as murine transformed non-malignant fibroblast cell line NIH/3T3 differed in their rates of COX-1 and COX-2 expression.

According to the obtained data, compound **5** did not disturb the viability of human melanoma A375 and human colon cancer cell lines HT29 and HCT116, human glioma cell line LN229 as well as the rat glioblastoma cell line C6. On the other hand, a slightly toxic effect in the high micromolar range could be detected for the human glioblastoma cell line U251 as well for the murine cell lines B16, B16–F10 and NIH/3T3. Overall, compound **5** was not cytotoxic against the tested cell lines.

### 2.5. Determination of GSM Activity

**Figure 1:**
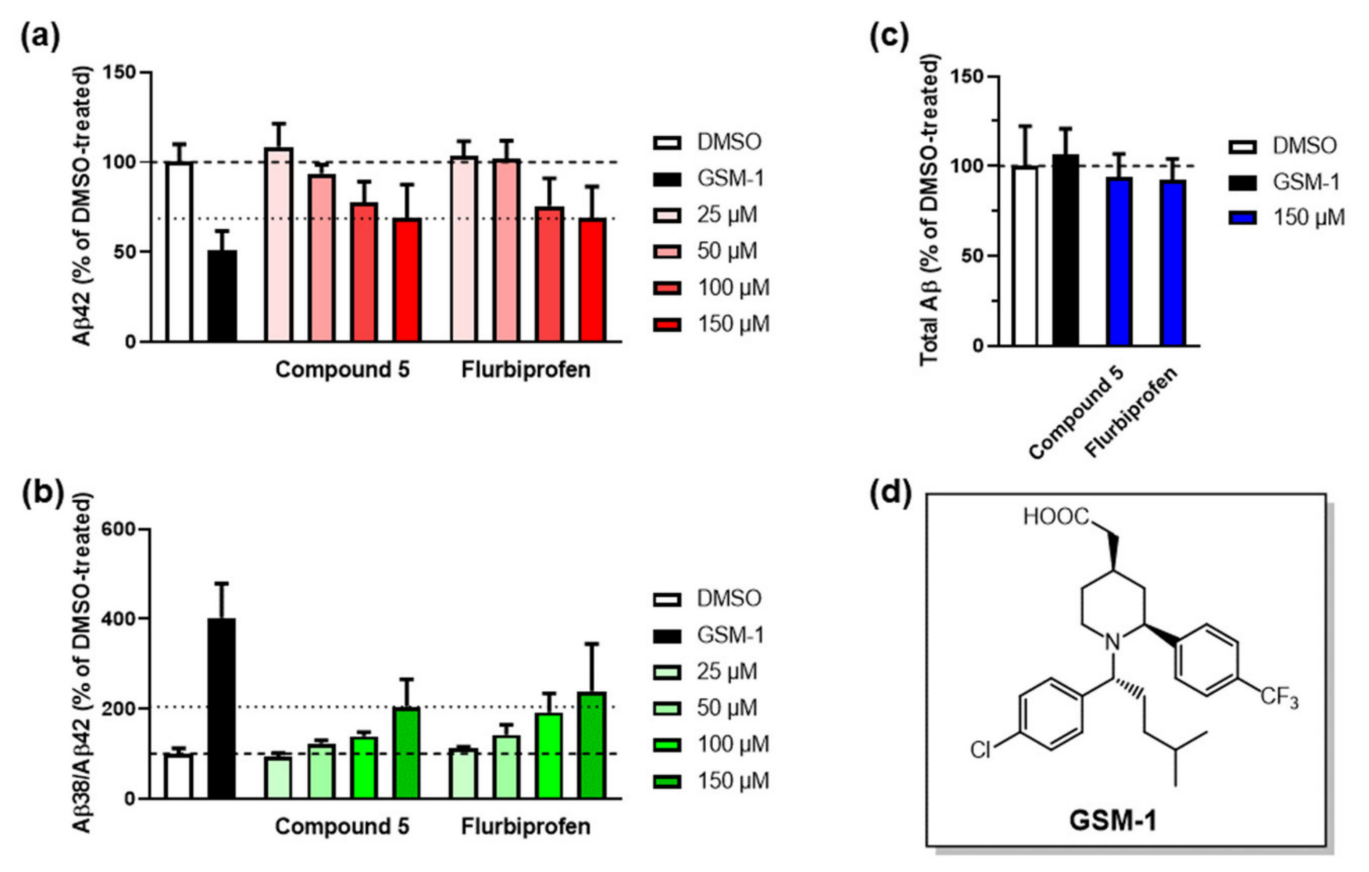
Compound **5** is a GSM with similar potency as flurbiprofen: (**a**) dose-dependent lowering of Aβ42, (**b**) dose-dependent increase in γ-secretase processivity, (**c**) stable maintenance of total Aβ levels (sum of measured Aβ38, Aβ40, and Aβ42) at the highest effective concentration. Data in (**a**–**c**) are represented as the mean ± SD (*n* = at least 6 biological replicates), the concentration of GSM-1 in all experiments was 1 µM. Aβ42 data in (**a**) are represented as ratio of total Aβ. (**d**) Structure of GSM-1 [56].

The γ-secretase modulator (GSM) activity of compound **5** was tested on human embryonic kidney (HEK) 293 cells stably overexpressing ”Swedish mutant” APP (HEK/sw), a well-established cell culture model to assess GSM activity [54,55]. The effect of compound **5** on Aβ production was analysed in comparison to flurbiprofen over a relevant and cell tolerable concentration range (Figure 1).

As shown in Figure 1a, a clear dose-dependent decrease in the pathogenic Aβ42 species was observed, which, as expected, was much weaker than that of 1 µM GSM-1 (Figure 1d), a potent acidic GSM used as the positive control [54]. As expected for a flurbiprofen-type GSM, compound **5** increased the ratio of short Aβ38 to the longer Aβ42 demonstrating that it elicited an increased processivity of the stepwise cleavage by γ-secretase (Figure 1b). The sum of all measured Aβ species (Aβ38, Aβ40 and Aβ42), as assessed at the most effective concentration of 150 µM, did not decrease significantly in comparison to the DMSO control, confirming that compound **5** is indeed a GSM and not a GSI (Figure 1c). The stable total Aβ levels also indicated that the substance was not harmful to the cells at the concentrations used in these assays. Comparison of these results to those of flurbiprofen suggest that the incorporation of a carborane moiety did neither reduce nor improve the modulatory activity of the compound.

## 3. Discussion

As *ortho*-carborane derivatives are readily deboronated under basic conditions [39,57], the more stable *meta*-carborane derivative was employed in our studies. Based on the findings of Wilkinson et al. [34], we wanted to ensure that the flurbiprofen derivative, already carrying one negative charge under physiological conditions, does not undergo deboronation with formation of a *nido*-carborane species, thus resulting in a dianion. A higher negative charge was expected to result in a decrease in logD_7.4_ below the desired range of one to three as well as an increase in electrostatic repulsion with the negatively charged cell membrane, thus making an active or passive transport through the membrane almost impossible.

In the GSM activity assays, racemic compound **5** was able to reduce the amount of produced extracellular Aβ42, thereby lowering the Aβ42:Aβ40 ratio. Together with the higher relative amount of Aβ38, this could lead to a reduction of amyloid plaque formation if taken up into the brain. The effect on Aβ production is comparable to that of flurbiprofen, but could not reach the activity of GSM-1 (Figure 1d), a highly potent second-generation GSM [54,55], indicating that introducing a carborane moiety did not result in improved GSM activity. The steric demand of the carborane moiety of compound **5** might be too high. In line with this, Peretto et al. observed that introduction of two chlorine atoms at positions 3′ and 5′ of flurbiprofen’s terminal ring, leading to an overall more globular steric demand, were not as beneficial as introduction at positions 3′ and 4′. An introduction of a chlorine atom at position 2’ blocked the formation of different conformations completely, resulting in a highly sterically demanding compound. This even led to an almost GSM-inactive derivative [20]. In this light, it is noteworthy that even with a sterically demanding carborane (van der Waals volumes: rotating benzene 102 Å^3^; *meta*-carborane 143 Å^3^) [58], it was possible to retain the GSM activity. The same could not be achieved by commonly substituted phenyl rings [20], underlining the value of carboranes as unique phenyl mimetics.

The inability of compound **5** to inhibit COX activity was unexpected. Instead, at least a minor inhibitory activity was suspected, due to the structural similarity to flurbiprofen. Although compound **5** was much more hydrophobic than racemic flurbiprofen (logD_7.4_ = 0.91), which might lead to solubility issues [46], the measured strong selectivity of indoborin for COX-2 proves that hydrophobicity should not affect the assay system itself. In addition, no precipitation was observed during the assay, and the carborane’s globular structure should sufficiently prevent π–π stacking. Thus, a simple solubility problem in the COX assay is unlikely but cannot be ruled out completely. However, this lack of COX inhibition could result in a compound that might not cause the cardiovascular and gastrointestinal side effects that are linked to inhibition of COX-1/2 by NSAIDs [59]. In this regard, the lack of activity by compound **5** on both COX isoforms is an important advantage over flurbiprofen.

The logD_7.4_ value of compound **5** could be successfully shifted to a range of logD_7.4_ = 1–3, logD_7.4_ ≥ 1.5, required for BBBcrossing [16,17]. The value is in a range comparable to drugs like dexamethasone (logD_7.4_ = 1.97 ± 0.03), a known BBB-crossing drug or the CNS-active anionic *nido*-carborane compound by Wilkinson et al. (logD_7.4_ = 1.44) [34,46,60]. Although negatively charged compounds, and carboxylic acids in particular, are generally considered to be poor BBB penetrators [16], it is our opinion that this does not necessarily hold true for carborane-derived compounds. Carboranes with their intrinsic high hydrophobicity might be able to sufficiently counterbalance the negative charge of a carboxylic acid. This was indicated by the increase in lipophilicity of compound **5** of around one order of magnitude. We achieved an increase in lipophilicity without the esterification of the carboxylic acid, which was shown to lead to an “inverse GSM” [21], making the resulting compound unsuitable for AD treatment. A higher lipophilicity could render the compound more susceptible to passive transport through the BBB. However, the general transport mechanism of flurbiprofen and other NSAIDs with carboxyl groups is not fully understood. Thus, involvement of active transporters, such as the monocarboxylate transporter (MCT) family or the human organic anion transporter (hOAT) family, is discussed [61,62]. In contrast to that, Parepally et al. could find no evidence of a saturable transport of flurbiprofen through the BBB, as it would be expected for a sole active transport mechanism [63]. Instead, they attributed the limitations of the transport through the BBB to the binding of flurbiprofen at bovine serum albumin (BSA), as rapid uptake of [^3^H]flurbiprofen was observed in vitro in the absence of BSA [63], since Goszczyński et al. could demonstrate that carboranes also show interactions with BSA [64], and the incorporation of *meta*-carborane into the flurbiprofen skeleton could even be of a disadvantage due to the higher plasma protein binding (PPB). However, it can be expected that replacement of the metabolically labile distal ring in flurbiprofen by the highly stable *meta*-carborane can prevent fast metabolic degradation at this ring and, thus, prolong the half-life of the drug, facilitating transfer through the BBB. This and the previous assumptions need to be further tested in future.

Another advantage of compound **5** is its low toxicity, which was determined by MTT and CV assays up to a concentration of 100 µM on several human and rodent cell lines. In addition, the influence of different COX-1 or COX-2 expression levels on the cell lines could not be observed. This is in accordance with the fact that no inhibition of the two COX isoforms could be determined. Of note, especially the high IC_50_ values against the human glioblastoma cell lines LN229 and U251 suggest the potential use of compound **5** as a CNS-active compound. In addition, no obvious toxicity was observed in human HEK cells up to 150 µM during the conducted GSM activity assays. Taken together, compound **5** provides a good toxicity profile for possible follow-up in vivo studies. In contrast, the carborane-analogues of the COX-2 selective inhibitors, celecoxib and rofecoxib, showed increased toxicity in the low micromolar range towards several cancer cell lines (A375, B16, B16–F10, and HCT116) [65,66]. This underlines that the site for introducing a carborane moiety has to be chosen carefully.

In summary, in a simple four-step synthetic sequence, a *meta*-carborane analogue of flurbiprofen was obtained in good overall yield. The derivatisation shifted the lipophilicity into a more favourable range required for BBB penetration. This could be achieved without diminishing the γ-secretase modulating activity. Especially, this last aspect of compound **5** underlines the benefit of the carborane moiety as phenyl mimetic in the development of potential AD drugs in future.

## 4. Material and Methods

### 4.1. Determination of COX Inhibition

COX inhibition potency against ovine COX-1 and human COX-2 was determined using the fluorescence-based COX assay “COX Fluorescent Inhibitor Screening Assay Kit” (catalog number 700100; Cayman Chemical Company, Ann Arbor, MI, USA) according to the manufacturer’s instructions as previously reported by us [44].

### 4.2. Determination of logD_7.4_

The logD measurements were conducted using the HPLC method originally described by Donovan et al. [45] and recently reported by us [44] on the HPLC system 1100 from Agilent Technologies with two different columns, a Chromolith SpeedROD RP-18E 50 from Merck and an Asahipak ODP-50 4B 4.6 × 50 from Shodex (Showa Denko Europe GmbH, Munich, Germany). In both cases, the eluent consisted of methanol and a 0.01 M phosphate buffer (pH = 7.4); the applied gradient was 0 min at 70% methanol, 10 min at 100% methanol, 20 min at 100% methanol at a flow rate of 0.6 mL/min.

### 4.3. Determination of Cytotoxicity

Cytotoxicity was determined by conducting MTT and CV assays using cell viability as an indicator [67]. All measurements were carried out in triplicates.

### 4.4. Determination of GSM Activity

Nearly confluent (80%) HEK/sw cells were treated with the indicated concentrations of compound **5** or flurbiprofen ((±)-2-fluoro-α-methyl-4-biphenylacetic acid; F8514, Sigma–Aldrich, St. Louis, MO, USA) as described before [55]. Cells treated with DMSO or 1 µM GSM-1 were used as negative and positive controls, respectively. After overnight treatment, the conditioned medium was collected, cleared by centrifugation (10 min, 4 °C, 16,000× *g*), and the levels of secreted Aβ38, Aβ40, and Aβ42 were analysed using an established Aβ species-specific sandwich immunoassay purchase from Meso Scale Discovery (MSD, Rockville, MD, USA) [54].

### 4.5. Synthetic Methods

#### 4.5.1. General Synthetic Information

All syntheses were carried out under nitrogen atmosphere using Schlenk technique. The solvents dichloromethane (DCM) and diethyl ether were dried with a solvent purification system SPS-800 Series from MBraun; carbon tetrachloride was dried over phosphorus pentoxide under nitrogen atmosphere. *meta*-Carborane was purchased from Katchem Ltd. (Prague, Czech Republic). All other chemicals were purchased from common suppliers. 9-Iodo-1,7-dicarba-*closo*-dodecaborane(12) (**1**) was prepared according to the literature [68,69].

NMR data were collected with either an Avance DRX-400 spectrometer (^1^H-NMR 400.13 MHz, ^13^C{^1^H}-NMR 100.63 MHz, ^11^B-NMR 128.38 MHz) or an Ascend-400 spectrometer (^1^H-NMR 400.16 MHz, ^13^C{^1^H}-NMR 100.63 MHz, ^11^B-NMR 128.38 MHz) from Bruker. ^1^H- and ^13^C-NMR spectra were referenced to tetramethylsilane and the ^11^B-NMR spectra to the Ξ scale [70]. Deuterated solvents were purchased from EURISO-TOP with a deuteration rate of 99.80%. All structural assignments of NMR signals were based on 2D NMR experiments, COSY, HSQC, HMQC and HMBC.

High-resolution ESI mass spectrometry was carried out on an Impact II from Bruker Daltonics and high-resolution EI mass spectrometry on an MAT 8230 from Finnigan (nowadays ThermoFisher Scientific, Waltham, MA, USA). The simulation of the mass spectra was conducted with a web-based program from Scientific Instrument Services Inc. (Palmer, MA, USA) [71].

The measurements of the IR spectra were carried out either on a Spektrum 2000 FT-IR from PerkinElmer (Waltham, MA, USA) with KBr pellets (KBr) or a Nicolette IS5 (ATR) from ThermoFisher (Waltham, MA, USA). The signal intensity was classified as weak (w), medium (m) or strong (s).

#### 4.5.2. Syntheses

**Scheme 2:**
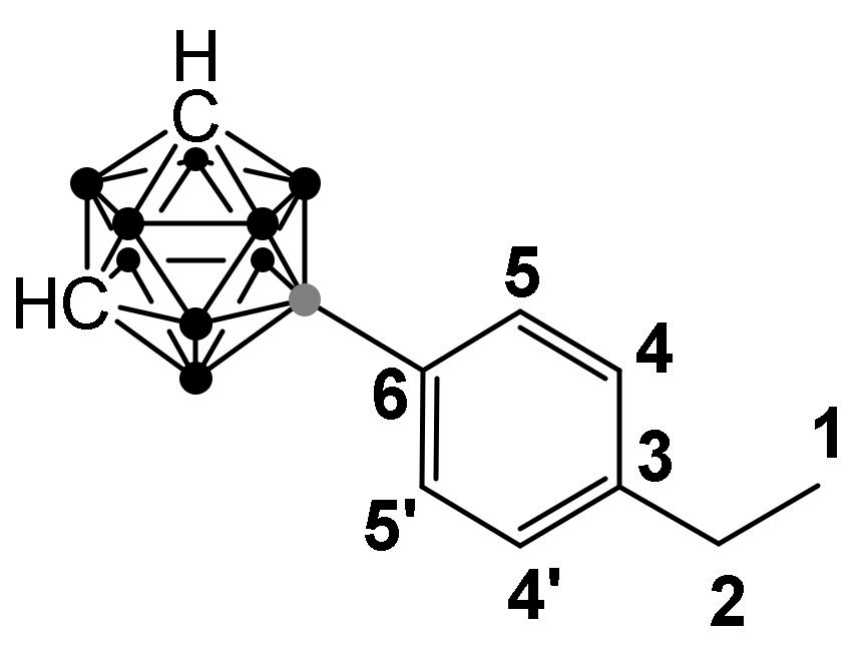
9-(4-Ethylphenyl)-1,7-dicarba-*closo*-dodecaborane(12) (**2**).

9-(4-Ethylphenyl)-1,7-dicarba-*closo*-dodecaborane(12) (**2**) (Scheme 2)

Magnesium turnings (1.84 g, 75.65 mmol, 7.0 eq) were heated to approximately 200 °C under high vacuum. After cooling to ambient temperature, 13 mL Et_2_O and 0.03 mL (0.065 g, 0.35 mmol, 0.03 eq) dibromoethane were added. Then, 10.00 g (54.04 mmol, 5.0 eq) 4-ethylbromobenzene in 60 mL Et_2_O was added dropwise at ambient temperature over 10 min. The mixture was refluxed for one hour, forming a deep red-brown suspension. The solid was filtered off and the resulting red-brown solution was added dropwise over 20 min to a solution of 2.92 g (10.81 mmol, 1.0 eq) 9-iodo-1,7-dicarba-*closo*-dodecaborane(12) (**1**) in 60 mL THF at 0 °C. After warming to ambient temperature, 50 mL THF, 0.38 g (0.54 mmol, 0.05 eq) [PdCl_2_(PPh_3_)_2_] and 0.10 g (0.54 mmol, 0.05 eq) CuI were added in one portion and the reaction mixture was refluxed for four days. The resulting black opaque mixture was cooled to 0 °C and the reaction quenched by adding 2 N HCl solution. The phases were separated, and the organic phase was washed once with 200 mL H_2_O/brine (3/1 (*v/v*)). The organic phase was then dried over MgSO_4_, filtered and the solvent removed under reduced pressure. The product was purified by column chromatography with *n*-hexane over silica, yielding 2.28 g (9.17 mmol, 85%) colourless crystals.

^1^H-NMR (CDCl_3_, 400 MHz): δ = 1.23 (3H, t, ^1^*J*_HH_ = 7.6 Hz, H-1), 1.50–3.50 (9H, br, BH), 2.62 (2H, q, ^1^*J*_HH_ = 7.6 Hz, H-2), 2.99 (2H, br s, H-carbon_cluster_), 7.12 (2H, m, H-4/4′), 7.44 (2H, d, ^1^*J*_HH_ = 7.6 Hz, H-5/5′) ppm. ^13^C{^1^H}-NMR (CDCl_3_, 100 MHz): δ = 15.4 (CH_3_, C-1), 28.6 (CH_2_, C-2), 54.1 (br, CH, C-cluster), 127.2 (CH, C-4/4′), 133.2 (CH, C-5/5′), 143.4 (C, C-3) ppm. The signal of the quaternary carbon atom C-6 could not be detected. ^11^B{^1^H}-NMR (CDCl_3_, 128 MHz): δ = −20.0 (1B, s, BH), −17.5 (1B, s, BH), −13.7 (2B, s, BH), −13.0 (2B, s, BH), −9.6 (1B, s, BH), −6.5 (2B, s, BH) and 0.5 (1B, s, BC) ppm. ^11^B-NMR (CDCl_3_, 128 MHz): δ = −20.0 (1B, d, ^1^*J*_BH_ = 181 Hz, BH), −17.5 (1B, d, ^1^*J*_BH_ = 181 Hz, BH), −15.5 to –11.5 (4B, br, BH), −9.6 (1B, d, ^1^*J*_BH_ = 154 Hz, BH), −6.5 (2B, d, ^1^*J*_BH_ = 162 Hz, BH) and 0.5 (1B, s, BC) ppm. HREIMS: *m/z* 248.257 (calculated for C_10_H_20_B_10_: *m/z* 248.257). IR: ṽ(ATR) = 3050 (s), 2962 (w), 2591 (s, BH), 1604 (w), 1507 (w), 1457 (w), 1435 (w), 1400 (w), 1229 (w), 1184 (w), 1158 (w), 1064 (m), 1028 (m), 988 (w), 867 (w), 845 (m), 829 (w) and 812 (w) cm^−1^.

**Scheme 3:**
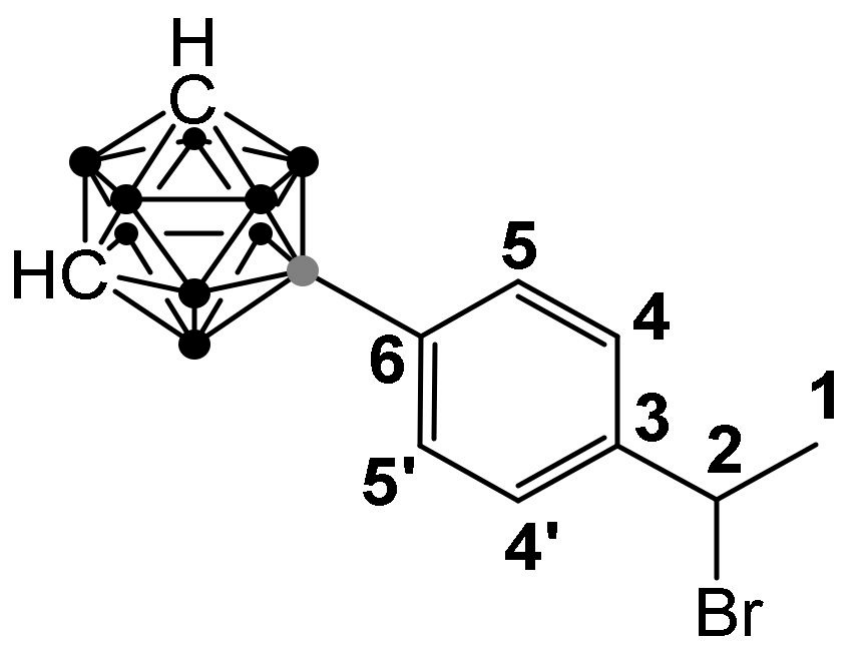
9-[4-(1-Bromoethyl)phenyl]-1,7-dicarba-*closo*-dodecaborane(12) (**3**).

9-[4-(1-Bromoethyl)phenyl]-1,7-dicarba-*closo*-dodecaborane(12) (**3**) (Scheme 3)

Compound **2** (0.30 g, 1.21 mmol, 1.0 eq) and 0.22 g (1.21 mmol, 1.0 eq) NBS were suspended in 20 mL CCl_4_. The mixture was irradiated with a 500 W halogen lamp for 45 min, causing the solvent to boil slightly. After cooling to ambient temperature, a precipitate formed, which was filtered off. The resulting clear solution was evaporated. The resulting oil (compound **3**) was directly used for the next step, without any further purification.

^1^H-NMR (CDCl_3_, 400 MHz): δ = 1.50–3.50 (9H, br, BH), 2.04 (3H, d, ^3^J_HH_ = 6.9 Hz, H-1), 3.01 (2H, br s, H-carbon_cluster_), 5.21 (1H, q, ^3^*J*_HH_ = 6.9 Hz, H-2), 7.34 (2H, d, ^3^*J*_HH_ = 8.1 Hz, H-4/4′), 7.44 (2H, d, ^1^*J*_HH_ = 7.9 Hz, H-5/5′) ppm. ^13^C{^1^H}-NMR (CDCl_3_, 100 MHz): δ = 26.7 (CH_3_, C-1), 50.0 (CH, C-2), 54.3 (CH, C_Cluster_), 125.9 (CH, C-4/4′), 133.5 (CH, C-5/5′) and 142.2 (C, C-3) ppm. The signal of the quaternary carbon C-6 could not be detected. ^11^B{^1^H}-NMR (CDCl_3_, 128 MHz): δ = −19.7 (1B, s, BH), −17.4 (1B, s, BH), −13.8 (2B, s, BH), −13.0 (2B, s, BH), −9.6 (1B, s, BH), −6.5 (2B, s, BH) and 0.2 (1B, s, BC) ppm. ^11^B-NMR (CDCl_3_, 128 MHz): δ = −19.7 (1B, d, ^1^*J*_BH_ = 186 Hz, BH), −17.4 (1B, d, ^1^*J*_BH_ = 181 Hz, BH), −15.5 to –11.5 (4B, br, BH), −9.6 (1B, d, ^1^*J*_BH_ = 152 Hz, BH), −6.5 (2B, d, ^1^*J*_BH_ = 163 Hz, BH) and 0.2 (1B, s, BC) ppm. HRESI: *m/z* 326.15803 (calculated for C_10_H_18_B_10_Br: *m/z* 326.15805).

**Scheme 4:**
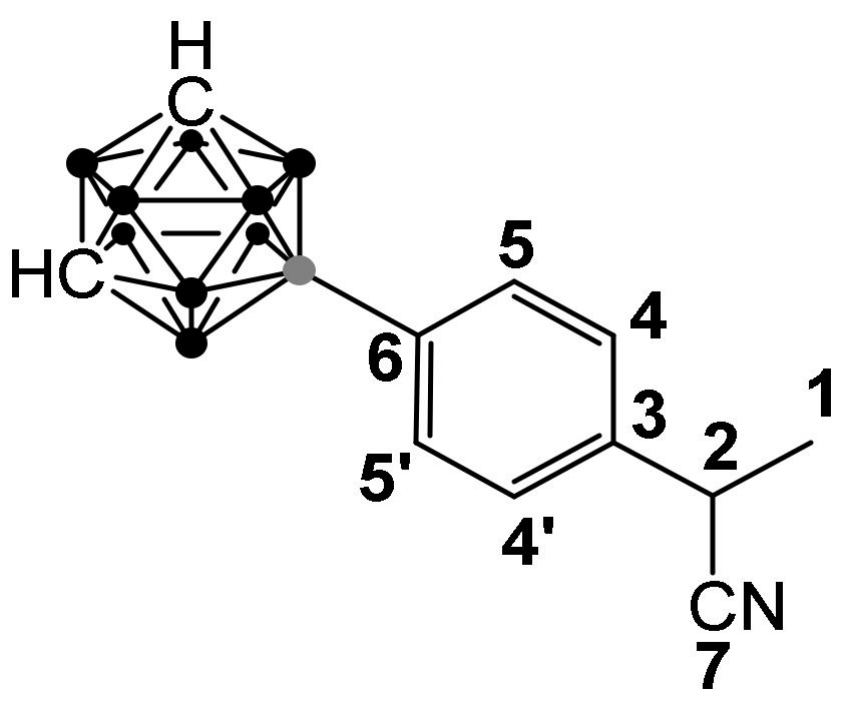
9-[4-(1-Cyanoethyl)phenyl]-1,7-dicarba-*closo*-dodecaborane(12) (**4**).

9-[4-(1-Cyanoethyl)phenyl]-1,7-dicarba-*closo*-dodecaborane(12) (**4**) (Scheme 4)

Crude **3** (0.08 g) and 0.19 g (1.88 mmol) trimethylsilylcyanide were dissolved in 15 mL DCM. The solution was cooled to 0 °C and 0.25 mL (0.56 g, 2.14 mmol) SnCl_4_ were added rapidly but dropwise. The resulting brown mixture was stirred at 0 °C for 20 min and then at ambient temperature for 2.5 h. The reaction was quenched with saturated Na_2_CO_3_ solution and the resulting mixture diluted with 50 mL DCM and 50 mL water. The two phases were separated and the aqueous phase was extracted with 50 mL DCM. The combined organic phases were dried over MgSO_4_, filtered and the solvent removed under reduced pressure. The product was purified by column chromatography, using *n*-hexane/ethylacetate mixtures as eluent. Yield 0.04 g (0.15 mmol, 99% over two steps).

^1^H-NMR (CDCl_3_, 400 MHz): δ = 1.60–3.50 (9H, br, BH), 1.63 (3H, d, ^3^*J*_HH_ = 7.3 Hz, H-1), 3.02 (2H, br s, H-carbon_cluster_), 3.86 (1H, q, ^3^*J*_HH_ = 7.3 Hz, H-2), 7.25 (2H, d, ^3^*J*_HH_ = 8.0 Hz, H-4/4′) and 7.52 (2H, d, ^3^*J*_HH_ = 7.7 Hz, H-5/5′) ppm. ^13^C{^1^H}-NMR (CDCl_3_, 100 MHz): δ = 21.4 (CH_3_, C-1), 31.1 (CH, C-2), 54.4 (CH, C_Cluster_), 121.7 (C, C-7), 125.9 (CH, C-4/4′), 133.9 (CH, C-5/5′) and 136.1 (C, C-3) ppm. The signal of the quaternary carbon C-6 could not be detected. ^11^B{^1^H}-NMR (CDCl_3_, 128 MHz): δ = −19.6 (1B, s, BH), −17.4 (1B, s, BH), −13.7 (2B, s, BH), −13.0 (2B, s, BH), −9.6 (1B, s, BH), −6.5 (2B, s, BH) and 0.2 (1B, s, BC) ppm. ^11^B-NMR (CDCl_3_, 128 MHz): δ = −19.6 (1B, d, ^1^*J*_BH_ = 180 Hz, BH), −17.4 (1B, d, ^1^*J*_BH_ = 181 Hz, BH), −13.7 (2B, d, ^1^*J*_BH_ = 167 Hz, BH), −13.0 (2B, d, ^1^*J*_BH_ = 159 Hz, BH), −9.6 (1B, d, ^1^*J*_BH_ = 152 Hz, BH), −6.5 (2B, d, ^1^*J*_BH_ = 163 Hz, BH) and 0.2 (1B, s, BC) ppm. HRESI: *m/z* 296.242 (calculated for C_11_H_19_B_10_NNa: *m/z* 296.241). IR: ṽ(KBr) = 3062 (s), 2995 (w), 2944 (w), 2878 (w), 2601 (s, BH), 2245 (w), 1510 (w), 1454 (w), 1398 (m), 1229 (m), 1159 (w), 1063 (m), 1032 (m), 991 (m), 945 (w), 879 (m), 839 (m), 813 (w) and 729 (m) cm^−1^.

**Scheme 5:**
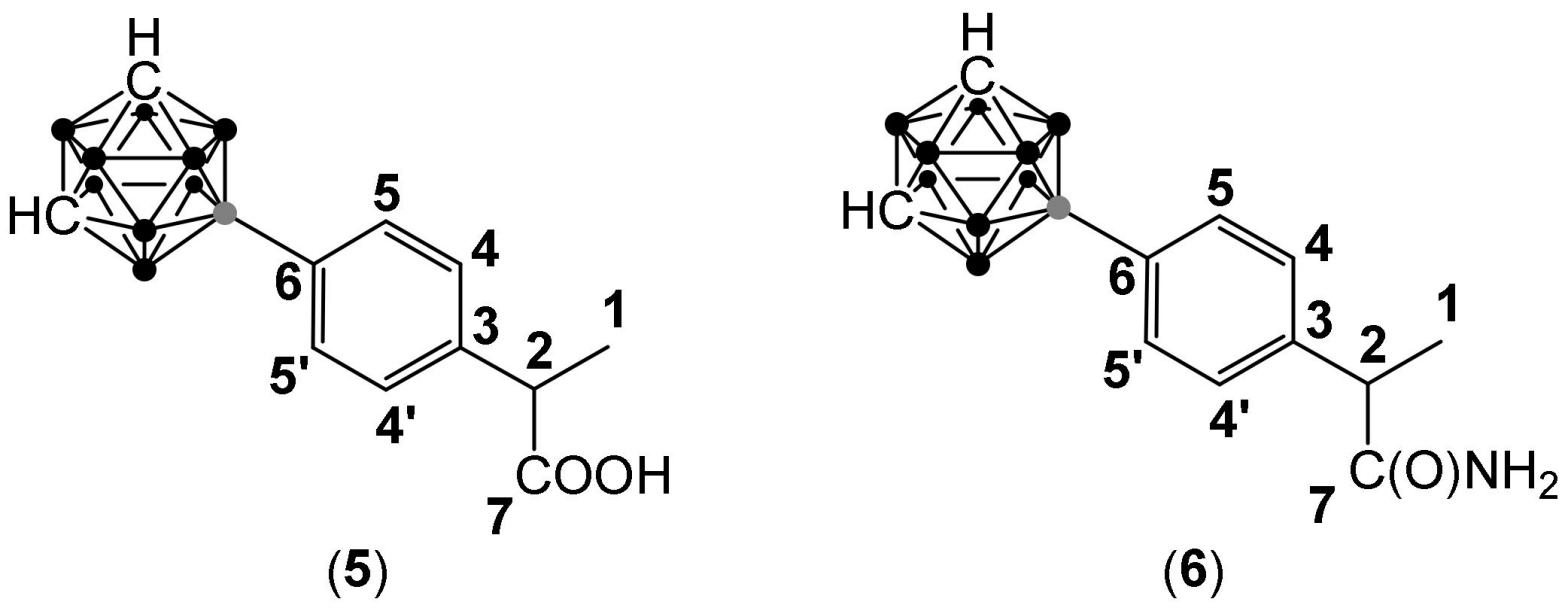
2-[4-(1,7-Dicarba-*closo*-dodecaboran-9-yl(12))phenyl]propionic acid (**5**) and 2-[4-(1,7-dicarba-*closo*-dodecaboran-9-yl(12))phenyl]propanamide (**6**).

2-[4-(1,7-Dicarba-*closo*-dodecaboran-9-yl(12))phenyl]propionic acid (**5**) and 2-[4-(1,7-dicarba-*closo*-dodecaboran-9-yl(12))phenyl]propanamide (**6**) (Scheme 5).

Compound 4 (0.017 g, 0.063 mmol, 1.0 eq) was suspended in 6 mL AcOH_conc._/HCl_aq., conc._ (5/1 (*v/v*)) and the resulting mixture was stirred under reflux for one hour and then at ambient temperature for three days. The clear solution was diluted with 50 mL water and extracted with 50 mL ethyl acetate. The phases were separated, and the organic phase was washed once with 50 mL water. The organic phase was dried over MgSO_4_, filtered and the solvent was removed under reduced pressure. The product was purified by column chromatography using *n*-hexane/ethyl acetate mixtures as eluent. Yield 0.007 g (0.023 mmol, 36%). In addition, 0.007 g (0.024 mmol, 38%) of the partially hydrolysed compounds, 2-[4-(1,7-dicarba-*closo*-dodecaboran-9-yl(12))phenyl]propanamide (**6**), could be isolated.

Analytical data of **5**

^1^H-NMR (CDCl_3_, 400 MHz): δ = 1.50 (3H, d, ^3^*J*_HH_ = 7.2 Hz, H-1), 1.50–3.50 (9H, br, BH), 3.00 (2H, br s, H-carbon_cluster_), 3.71 (1H, q, ^3^*J*_HH_ = 7.2 Hz, H-2), 7.22 (2H, d, ^3^*J*_HH_ = 8.0 Hz, H-4/4′), 7.50 (2H, d, ^3^*J*_HH_ = 7.9 Hz, H-5/5′) and 10.5 (1H, br s, COOH) ppm. ^13^C{^1^H}-NMR (CDCl_3_, 100 MHz): δ = 18.1 (CH_3_, C-1), 45.1 (CH, C-2), 54.2 (CH, C_Cluster_), 126.8 (CH, C-4/4′), 133.5 (CH, C-5/5′), 138.8 (C, C-3) and 180.1 (C-7) ppm. The signal of the quaternary carbon C-6 could not be detected. ^11^B{^1^H}-NMR (CDCl_3_, 128 MHz): δ = −19.7 (1B, s, BH), −17.4 (1B, s, BH), −13.8 (2B, s, BH), −13.0 (2B, s, BH), −9.6 (1B, s, BH), −6.5 (2B, s, BH) and 0.31 (1B, s, BC) ppm. ^11^B-NMR (CDCl_3_, 128 MHz): δ = −19.7 (1B, d, ^1^*J*_BH_ = 195 Hz, BH), −17.5 (1B, d, ^1^*J*_BH_ = 182 Hz, BH), −15.5 to –11.0 (4B, br, BH), −9.6 (1B, d, ^1^*J*_BH_ = 151 Hz, BH), −6.5 (2B, d, ^1^J_BH_ = 163 Hz, BH) and 0.31 (1B, s, BC) ppm. HRESI: *m/z* 315.237 (calculated for C_11_H_20_B_10_NaO_2_: *m/z* 315.236). IR: ṽ(KBr) = 1377 (w), 1283 (m), 1252 (m), 1228 (s), 1156 (w), 1071 (m), 1031 (w), 921 (m), 867 (s), 796 (m), 740 (w), 726 (m) and 668 (m) cm^−1^.

Analytical data of **6**

^1^H-NMR (CDCl_3_, 400 MHz): δ = 1.52 (3H, d, ^3^J_HH_ = 7.2 Hz, H-1), 1.50–3.50 (9H, br, BH), 3.02 (br s, 2H, H-carbon_Cluster_), 3.58 (1H, q, ^3^*J*_HH_ = 7.2 Hz, H-2), 5.34 (2H, br s, NH_2_), 7.21 (2H, d, ^3^*J*_HH_ = 8.0 Hz, H-4/4′) and 7.49 (2H, d, ^3^*J*_HH_ = 7.7 Hz, H-5/5′) ppm. ^13^C{^1^H}-NMR (CDCl_3_, 100 MHz): δ = 18.2 (CH_3_, C-1), 46.4 (CH, C-2), 54.3 (CH, C_Cluster_), 126.9 (CH, C-4/4′), 133.8 (CH, C-5/5′), 140.2 (C, C-3) and 176.7 (C-7) ppm. The signal of the quaternary carbon C-6 could not be detected. ^11^B{^1^H}-NMR (CDCl_3_, 128 MHz): δ = −19.7 (1B, s, BH), −17.4 (1B, s, BH), −13.8 (2B, s, BH), −13.0 (2B, s, BH), −9.6 (1B, s, BH), −6.5 (2B, s, BH) and 0.23 (1B, s, BC) ppm. ^11^B-NMR (CDCl_3_, 128 MHz): δ = −19.7 (1B, d, ^1^*J*_BH_ = 178 Hz, BH), −17.5 (1B, d, ^1^*J*_BH_ = 181 Hz, BH), −15.5 to –11.0 (4B, br, BH), −9.6 (1B, d, ^1^*J*_BH_ = 151 Hz, BH), −6.5 (2B, d, ^1^*J*_BH_ = 163 Hz, BH) and 0.23 (1B, s, BC) ppm. HRESI: *m/z* 314.252 (calculated for C_11_H_21_B_10_NNaO: *m/z* 314.252).

## Figures and Tables

**Table 1:** IC_50_ values of compound **5** and *nido*-indoborin for the inhibition of COX-1 and COX-2. Tested with the commercial “COX Fluorescent Inhibitor Screening Assay Kit” from Cayman Chemical Company.

Enzyme	Compound 5	*nido*-Indoborin		Celecoxib
		Measured	Reported [39]	
COX-1	>100 µM	>100 µM	>4 µM	100 µM
COX-2	>100 µM	0.91 µM	0.051 µM	0.04 ± 0.02 µM

**Table 2:** LogD_7.4_ of compound **5** and *nido*-indoborin. Method 1: Chromolith Speed ROD RP-18E 50 from Merck, method 2: Asahipak ODP-50 4B 4.6 × 50 from Shodex.

Method	Compound 5	*nido*-Indoborin	Flurbiprofen [46] (Shake Flask)
1	2.00	1.94	0.91
2	1.86	2.05	

**Table 3:** Cytotoxicity of compound **5** presented as IC_50_ values. All values are given in (mean ± SD) µM. A375: human melanoma cancer cell line expressing COX-1 and COX-2 [47]. HCT116: human colon cancer cell line expressing COX-1 but not COX-2 [48]. HT29: human colon cancer cell line expressing COX-1 and COX-2 [48]. LN229 and U251: human glioblastoma cell lines non-expressing COX-2 [49,50]. NIH/3T3: mouse fibroblast cell line with expression of COX-1 and low expression of COX-2 [51]. B16 and its metastatic clone, B16–F10, murine melanoma cell lines, expressing both COX-1 and COX-2 [52]. C6: rat glioma cell line expressing both COX-1 and COX-2 [53].

Human			Rodent		
Cell Line	MTT	CV	Cell Line	MTT	CV
A375	96 ± 5.9	>100	B16	79 ± 2.4	93.7 ± 3.8
HCT116	>100	>100	B16–F10	74.7 ± 0.9	95 ± 4.6
HT29	>100	>100	NIH/3T3	90 ± 10	>100
LN229	95 ± 7.1	93.3 ± 9.5	C6	92.8 ± 4.8	99.4 ± 0.8
U251	77.5 ± 7.2	90.1 ± 7.1			

## Data Availability

The data presented in this study is contained within the article or is available on request from the corresponding author.

## Sample Availability

Samples of the compounds are not available from the authors.
